# Supplementary material for: Iterative improvement in the automatic modular design of robot swarms
Source: PeerJ Comput Sci. 2020 Dec 7;6:e322. doi: 10.7717/peerj-cs.322 (PMC7924708; doi:10.7717/peerj-cs.322)
Supplement: Supplemental Information 3 [file peerj-cs-06-322-s003.zip › argos3/doc/api/standalone/a00646_source.html]

ARGoS: plugins/simulator/visualizations/qt-opengl/qtopengl\_lua\_main\_window.cpp Source File


- Main Page
- Related Pages
- Namespaces
- Classes
- Files

- File List
- File Members

# plugins/simulator/visualizations/qt-opengl/qtopengl\_lua\_main\_window.cpp

Go to the documentation of this file.

```
00001 
00006 #include "qtopengl_lua_main_window.h"
00007 #include "qtopengl_lua_editor.h"
00008 #include "qtopengl_lua_find_dialog.h"
00009 #include "qtopengl_lua_statetree_model.h"
00010 #include "qtopengl_main_window.h"
00011 #include "qtopengl_widget.h"
00012 
00013 #include <argos3/core/config.h>
00014 #include <argos3/core/wrappers/lua/lua_controller.h>
00015 #include <argos3/core/simulator/simulator.h>
00016 #include <argos3/core/simulator/space/space.h>
00017 #include <argos3/core/simulator/entity/composable_entity.h>
00018 #include <argos3/core/simulator/entity/controllable_entity.h>
00019 #include <argos3/core/wrappers/lua/lua_utility.h>
00020 
00021 #include <QApplication>
00022 #include <QDockWidget>
00023 #include <QFileDialog>
00024 #include <QHeaderView>
00025 #include <QMenu>
00026 #include <QMenuBar>
00027 #include <QMessageBox>
00028 #include <QProcess>
00029 #include <QSettings>
00030 #include <QStatusBar>
00031 #include <QTemporaryFile>
00032 #include <QTextStream>
00033 #include <QToolBar>
00034 #include <QTableWidget>
00035 #include <QTreeView>
00036 
00037 namespace argos {
00038 
00039    /****************************************/
00040    /****************************************/
00041 
00042    static QString SCRIPT_TEMPLATE =
00043       "-- Use Shift + Click to select a robot\n"
00044       "-- When a robot is selected, its variables appear in this editor\n\n"
00045       "-- Use Ctrl + Click (Cmd + Click on Mac) to move a selected robot to a different location\n\n\n\n"
00046       "-- Put your global variables here\n\n\n\n"
00047       "--[[ This function is executed every time you press the 'execute' button ]]\n"
00048       "function init()\n"
00049       "   -- put your code here\n"
00050       "end\n\n\n\n"
00051       "--[[ This function is executed at each time step\n"
00052       "     It must contain the logic of your controller ]]\n"
00053       "function step()\n"
00054       "   -- put your code here\n"
00055       "end\n\n\n\n"
00056       "--[[ This function is executed every time you press the 'reset'\n"
00057       "     button in the GUI. It is supposed to restore the state\n"
00058       "     of the controller to whatever it was right after init() was\n"
00059       "     called. The state of sensors and actuators is reset\n"
00060       "     automatically by ARGoS. ]]\n"
00061       "function reset()\n"
00062       "   -- put your code here\n"
00063       "end\n\n\n\n"
00064       "--[[ This function is executed only once, when the robot is removed\n"
00065       "     from the simulation ]]\n"
00066       "function destroy()\n"
00067       "   -- put your code here\n"
00068       "end\n";
00069 
00070    /****************************************/
00071    /****************************************/
00072 
00073    CQTOpenGLLuaMainWindow::CQTOpenGLLuaMainWindow(CQTOpenGLMainWindow* pc_parent) :
00074       QMainWindow(pc_parent),
00075       m_pcMainWindow(pc_parent),
00076       m_pcStatusbar(NULL),
00077       m_pcCodeEditor(NULL),
00078       m_pcFindDialog(NULL),
00079       m_pcLuaMessageTable(NULL) {
00080       /* Add a status bar */
00081       m_pcStatusbar = new QStatusBar(this);
00082       setStatusBar(m_pcStatusbar);
00083       /* Create the Lua message table */
00084       CreateLuaMessageTable();
00085       /* Populate list of Lua controllers */
00086       PopulateLuaControllers();
00087       /* Create the Lua state docks */
00088       CreateLuaStateDocks();
00089       /* Create editor */
00090       CreateCodeEditor();
00091       /* Create actions */
00092       CreateFileActions();
00093       CreateEditActions();
00094       CreateCodeActions();
00095       /* Set empty file */
00096       SetCurrentFile("");
00097       /* Read settings */
00098       ReadSettings();
00099    }
00100 
00101    /****************************************/
00102    /****************************************/
00103 
00104    CQTOpenGLLuaMainWindow::~CQTOpenGLLuaMainWindow() {
00105       WriteSettings();
00106    }
00107 
00108    /****************************************/
00109    /****************************************/
00110 
00111    void CQTOpenGLLuaMainWindow::New() {
00112       if(MaybeSave()) {
00113          m_pcCodeEditor->setPlainText(SCRIPT_TEMPLATE);
00114          SetCurrentFile("");
00115       }
00116    }
00117 
00118    /****************************************/
00119    /****************************************/
00120 
00121    void CQTOpenGLLuaMainWindow::Open() {
00122       if(MaybeSave()) {
00123          QString strNewFileName =
00124             QFileDialog::getOpenFileName(this,
00125                                          tr("Open File"),
00126                                          "",
00127                                          "Lua Files (*.lua)");
00128          if (!strNewFileName.isEmpty()) {
00129             OpenFile(strNewFileName);
00130          }
00131       }
00132    }
00133 
00134    /****************************************/
00135    /****************************************/
00136 
00137    void CQTOpenGLLuaMainWindow::OpenRecentFile() {
00138       QAction* pcAction = qobject_cast<QAction*>(sender());
00139       if (pcAction) {
00140          OpenFile(pcAction->data().toString());
00141       }
00142    }
00143 
00144    /****************************************/
00145    /****************************************/
00146 
00147    bool CQTOpenGLLuaMainWindow::Save() {
00148       if (m_strFileName.isEmpty()) {
00149          return SaveAs();
00150       } else {
00151          return SaveFile(m_strFileName);
00152       }
00153    }
00154 
00155    /****************************************/
00156    /****************************************/
00157 
00158    bool CQTOpenGLLuaMainWindow::SaveAs() {
00159       QString strNewFileName =
00160          QFileDialog::getSaveFileName(this,
00161                                       tr("Save File"),
00162                                       "",
00163                                       "Lua Files (*.lua)");
00164       if (strNewFileName.isEmpty())
00165          return false;
00166       return SaveFile(strNewFileName);
00167    }
00168 
00169    /****************************************/
00170    /****************************************/
00171 
00172    static QString DetectLuaC() {
00173       QProcess cLuaCompiler;
00174       cLuaCompiler.start("luac", QStringList() << "-v");
00175       /* First, try to execute luac */
00176       if(!cLuaCompiler.waitForStarted()) {
00177          /* luac is not installed */
00178          return "";
00179       }
00180       /* luac is installed, but is it the right version? */
00181       cLuaCompiler.waitForFinished();
00182       if(QString(cLuaCompiler.readAllStandardOutput()).mid(4,3) == "5.2") {
00183          return "luac";
00184       }
00185       cLuaCompiler.start("luac5.2", QStringList() << "-v");
00186       if(!cLuaCompiler.waitForStarted()) {
00187          /* luac52 is not installed */
00188          return "";
00189       }
00190       else {
00191          /* luac52 is installed */
00192          cLuaCompiler.waitForFinished();
00193          return "luac5.2";
00194       }
00195    }
00196 
00197    void CQTOpenGLLuaMainWindow::Execute() {
00198       /* Save script */
00199       Save();
00200       /* Change cursor */
00201       QApplication::setOverrideCursor(Qt::WaitCursor);
00202       /* Stop simulation */
00203       m_pcMainWindow->SuspendExperiment();
00204       /* Clear the message table */
00205       m_pcLuaMessageTable->clearContents();
00206       m_pcLuaMessageTable->setRowCount(1);
00207       /* Create temporary file to contain the bytecode */
00208       QTemporaryFile cByteCode;
00209       if(! cByteCode.open()) {
00210          SetMessage(0, "ALL", "Can't create bytecode file.");
00211          QApplication::restoreOverrideCursor();
00212          return;
00213       }
00214       /*
00215        * Compile script
00216        */
00217       /* Check for luac 5.2 */
00218       QString cLuaC = DetectLuaC();
00219       if(cLuaC == "") {
00220          /* luac 5.2 not found, fall back to sending the script directly to robots */
00221          for(size_t i = 0; i < m_vecControllers.size(); ++i) {
00222             m_vecControllers[i]->SetLuaScript(m_strFileName.toStdString());
00223          }
00224       }
00225       else {
00226          QProcess cLuaCompiler;
00227          cLuaCompiler.start(cLuaC, QStringList() << "-o" << cByteCode.fileName() << m_strFileName);
00228          if(! cLuaCompiler.waitForFinished()) {
00229             SetMessage(0, "ALL", QString(cLuaCompiler.readAllStandardError()));
00230             QApplication::restoreOverrideCursor();
00231             return;
00232          }
00233          if(cLuaCompiler.exitCode() != 0) {
00234             SetMessage(0, "ALL", QString(cLuaCompiler.readAllStandardError()));
00235             QApplication::restoreOverrideCursor();
00236             return;
00237          }
00238          SetMessage(0, "ALL", "Compilation successful.");
00239          /* Set the script for all the robots */
00240          for(size_t i = 0; i < m_vecControllers.size(); ++i) {
00241             m_vecControllers[i]->SetLuaScript(cByteCode.fileName().toStdString());
00242          }
00243       }
00244       /* Update Lua state if visible */
00245       if(m_pcLuaVariableDock->isVisible()) {
00246          static_cast<CQTOpenGLLuaStateTreeModel*>(m_pcLuaVariableTree->model())->SetLuaState(
00247             m_vecControllers[m_unSelectedRobot]->GetLuaState());
00248       }
00249       if(m_pcLuaFunctionDock->isVisible()) {
00250          static_cast<CQTOpenGLLuaStateTreeModel*>(m_pcLuaFunctionTree->model())->SetLuaState(
00251             m_vecControllers[m_unSelectedRobot]->GetLuaState());
00252       }
00253       /* Resume simulation */
00254       m_pcMainWindow->ResumeExperiment();
00255       QApplication::restoreOverrideCursor();
00256       statusBar()->showMessage(tr("Execution started"), 2000);
00257    }
00258 
00259    /****************************************/
00260    /****************************************/
00261 
00262    void CQTOpenGLLuaMainWindow::Find() {
00263       if(! m_pcFindDialog) {
00264          m_pcFindDialog = new CQTOpenGLLuaFindDialog(this);
00265       }
00266       m_pcFindDialog->show();
00267    }
00268 
00269    /****************************************/
00270    /****************************************/
00271 
00272    void CQTOpenGLLuaMainWindow::CodeModified() {
00273       setWindowModified(m_pcCodeEditor->document()->isModified());
00274    }
00275 
00276    /****************************************/
00277    /****************************************/
00278 
00279    bool CQTOpenGLLuaMainWindow::MaybeSave() {
00280       if(m_pcCodeEditor->document()->isModified()) {
00281          QMessageBox::StandardButton tReply;
00282          tReply = QMessageBox::warning(this, tr("ARGoS v" ARGOS_VERSION "-" ARGOS_RELEASE " - Lua Editor"),
00283                                        tr("The document has been modified.\n"
00284                                           "Do you want to save your changes?"),
00285                                        QMessageBox::Save | QMessageBox::Discard | QMessageBox::Cancel);
00286          if (tReply == QMessageBox::Save)
00287             return Save();
00288          else if (tReply == QMessageBox::Cancel)
00289             return false;
00290       }
00291       return true;
00292    }
00293 
00294    /****************************************/
00295    /****************************************/
00296 
00297    void CQTOpenGLLuaMainWindow::PopulateLuaControllers() {
00298       /* Get list of controllable entities */
00299       CSpace& cSpace = CSimulator::GetInstance().GetSpace();
00300       CSpace::TMapPerType& tControllables = cSpace.GetEntitiesByType("controller");
00301       /* Go through them and keep a pointer to each Lua controller */
00302       for(CSpace::TMapPerType::iterator it = tControllables.begin();
00303           it != tControllables.end();
00304           ++it) {
00305          /* Try to convert the controller into a Lua controller */
00306          CControllableEntity* pcControllable = any_cast<CControllableEntity*>(it->second);
00307          CLuaController* pcLuaController = dynamic_cast<CLuaController*>(&(pcControllable->GetController()));
00308          if(pcLuaController) {
00309             /* Conversion succeeded, add to indices */
00310             m_vecControllers.push_back(pcLuaController);
00311             m_vecRobots.push_back(&pcControllable->GetParent());
00312          }
00313          else {
00314             LOGERR << "[WARNING] Entity \""
00315                    << pcControllable->GetParent().GetId()
00316                    << "\" does not have a Lua controller associated"
00317                    << std::endl;
00318          }
00319       }
00320    }
00321 
00322    /****************************************/
00323    /****************************************/
00324 
00325    void CQTOpenGLLuaMainWindow::ReadSettings() {
00326       QSettings cSettings;
00327       cSettings.beginGroup("LuaEditor");
00328       resize(cSettings.value("size", QSize(640,480)).toSize());
00329       move(cSettings.value("position", QPoint(0,0)).toPoint());
00330       cSettings.endGroup();
00331    }
00332 
00333    /****************************************/
00334    /****************************************/
00335 
00336    void CQTOpenGLLuaMainWindow::WriteSettings() {
00337       QSettings cSettings;
00338       cSettings.beginGroup("LuaEditor");
00339       cSettings.setValue("size", size());
00340       cSettings.setValue("position", pos());
00341       cSettings.endGroup();
00342    }
00343 
00344    /****************************************/
00345    /****************************************/
00346 
00347    void CQTOpenGLLuaMainWindow::CreateCodeEditor() {
00348       /* Create code editor */
00349       m_pcCodeEditor = new CQTOpenGLLuaEditor(this);
00350       setCentralWidget(m_pcCodeEditor);
00351       m_pcCodeEditor->setPlainText(SCRIPT_TEMPLATE);
00352       /* Connect stuff */
00353       connect(m_pcCodeEditor->document(), SIGNAL(contentsChanged()),
00354               this, SLOT(CodeModified()));
00355       connect(&(m_pcMainWindow->GetOpenGLWidget()), SIGNAL(StepDone(int)),
00356               this, SLOT(CheckLuaStatus(int)));
00357    }
00358 
00359    /****************************************/
00360    /****************************************/
00361 
00362    void CQTOpenGLLuaMainWindow::CreateLuaMessageTable() {
00363       m_pcLuaMsgDock = new QDockWidget(tr("Messages"), this);
00364       m_pcLuaMsgDock->setObjectName("LuaMessageDock");
00365       m_pcLuaMsgDock->setFeatures(QDockWidget::DockWidgetMovable | QDockWidget::DockWidgetFloatable);
00366       m_pcLuaMsgDock->setAllowedAreas(Qt::LeftDockWidgetArea | Qt::RightDockWidgetArea | Qt::BottomDockWidgetArea);
00367       m_pcLuaMessageTable = new QTableWidget();
00368       m_pcLuaMessageTable->setColumnCount(3);
00369       QStringList listHeaders;
00370       listHeaders << tr("Robot")
00371                   << tr("Line")
00372                   << tr("Message");
00373       m_pcLuaMessageTable->setHorizontalHeaderLabels(listHeaders);
00374       m_pcLuaMessageTable->horizontalHeader()->setStretchLastSection(true);
00375       m_pcLuaMessageTable->setSelectionBehavior(QAbstractItemView::SelectRows);
00376       m_pcLuaMessageTable->setSelectionMode(QAbstractItemView::SingleSelection);
00377       m_pcLuaMsgDock->setWidget(m_pcLuaMessageTable);
00378       addDockWidget(Qt::BottomDockWidgetArea, m_pcLuaMsgDock);
00379       connect(m_pcLuaMessageTable, SIGNAL(itemSelectionChanged()),
00380               this, SLOT(HandleMsgTableSelection()));
00381       m_pcLuaMsgDock->hide();
00382    }
00383 
00384    /****************************************/
00385    /****************************************/
00386 
00387    void CQTOpenGLLuaMainWindow::CreateLuaStateDocks() {
00388       /* Variable tree dock */
00389       m_pcLuaVariableDock = new QDockWidget(tr("Variables"), this);
00390       m_pcLuaVariableDock->setObjectName("LuaVariableDock");
00391       m_pcLuaVariableDock->setFeatures(QDockWidget::DockWidgetMovable | QDockWidget::DockWidgetFloatable);
00392       m_pcLuaVariableDock->setAllowedAreas(Qt::LeftDockWidgetArea | Qt::RightDockWidgetArea | Qt::BottomDockWidgetArea);
00393       m_pcLuaVariableTree = new QTreeView();
00394       m_pcLuaVariableDock->setWidget(m_pcLuaVariableTree);
00395       addDockWidget(Qt::LeftDockWidgetArea, m_pcLuaVariableDock);
00396       m_pcLuaVariableDock->hide();
00397       /* Function tree dock */
00398       m_pcLuaFunctionDock = new QDockWidget(tr("Functions"), this);
00399       m_pcLuaFunctionDock->setObjectName("LuaFunctionDock");
00400       m_pcLuaFunctionDock->setFeatures(QDockWidget::DockWidgetMovable | QDockWidget::DockWidgetFloatable);
00401       m_pcLuaFunctionDock->setAllowedAreas(Qt::LeftDockWidgetArea | Qt::RightDockWidgetArea | Qt::BottomDockWidgetArea);
00402       m_pcLuaFunctionTree = new QTreeView();
00403       m_pcLuaFunctionDock->setWidget(m_pcLuaFunctionTree);
00404       addDockWidget(Qt::LeftDockWidgetArea, m_pcLuaFunctionDock);
00405       m_pcLuaFunctionDock->hide();
00406       /* Connect stuff */
00407       connect(&(m_pcMainWindow->GetOpenGLWidget()), SIGNAL(EntitySelected(size_t)),
00408               this, SLOT(HandleEntitySelection(size_t)));
00409       connect(&(m_pcMainWindow->GetOpenGLWidget()), SIGNAL(EntityDeselected(size_t)),
00410               this, SLOT(HandleEntityDeselection(size_t)));
00411    }
00412 
00413    /****************************************/
00414    /****************************************/
00415 
00416    void CQTOpenGLLuaMainWindow::CreateFileActions() {
00417       QIcon cFileNewIcon;
00418       cFileNewIcon.addPixmap(QPixmap(m_pcMainWindow->GetIconDir() + "/new.png"));
00419       m_pcFileNewAction = new QAction(cFileNewIcon, tr("&New"), this);
00420       m_pcFileNewAction->setToolTip(tr("Create a new file"));
00421       m_pcFileNewAction->setStatusTip(tr("Create a new file"));
00422       m_pcFileNewAction->setShortcut(QKeySequence::New);
00423       connect(m_pcFileNewAction, SIGNAL(triggered()),
00424               this, SLOT(New()));
00425       QIcon cFileOpenIcon;
00426       cFileOpenIcon.addPixmap(QPixmap(m_pcMainWindow->GetIconDir() + "/open.png"));
00427       m_pcFileOpenAction = new QAction(cFileOpenIcon, tr("&Open..."), this);
00428       m_pcFileOpenAction->setToolTip(tr("Open a file"));
00429       m_pcFileOpenAction->setStatusTip(tr("Open a file"));
00430       m_pcFileOpenAction->setShortcut(QKeySequence::Open);
00431       connect(m_pcFileOpenAction, SIGNAL(triggered()),
00432               this, SLOT(Open()));
00433       for (int i = 0; i < MAX_RECENT_FILES; ++i) {
00434          m_pcFileOpenRecentAction[i] = new QAction(this);
00435          m_pcFileOpenRecentAction[i]->setVisible(false);
00436          connect(m_pcFileOpenRecentAction[i], SIGNAL(triggered()),
00437                  this, SLOT(OpenRecentFile()));
00438       }
00439       QIcon cFileSaveIcon;
00440       cFileSaveIcon.addPixmap(QPixmap(m_pcMainWindow->GetIconDir() + "/save.png"));
00441       m_pcFileSaveAction = new QAction(cFileSaveIcon, tr("&Save"), this);
00442       m_pcFileSaveAction->setToolTip(tr("Save the current file"));
00443       m_pcFileSaveAction->setStatusTip(tr("Save the current file"));
00444       m_pcFileSaveAction->setShortcut(QKeySequence::Save);
00445       connect(m_pcFileSaveAction, SIGNAL(triggered()),
00446               this, SLOT(Save()));
00447       QIcon cFileSaveAsIcon;
00448       cFileSaveAsIcon.addPixmap(QPixmap(m_pcMainWindow->GetIconDir() + "/saveas.png"));
00449       m_pcFileSaveAsAction = new QAction(cFileSaveAsIcon, tr("S&ave as..."), this);
00450       m_pcFileSaveAsAction->setToolTip(tr("Save the current file under a new name"));
00451       m_pcFileSaveAsAction->setStatusTip(tr("Save the current file under a new name"));
00452       m_pcFileSaveAsAction->setShortcut(QKeySequence::SaveAs);
00453       connect(m_pcFileSaveAsAction, SIGNAL(triggered()),
00454               this, SLOT(SaveAs()));
00455       QMenu* pcMenu = menuBar()->addMenu(tr("&File"));
00456       pcMenu->addAction(m_pcFileNewAction);
00457       pcMenu->addSeparator();
00458       pcMenu->addAction(m_pcFileOpenAction);
00459       pcMenu->addSeparator();
00460       pcMenu->addAction(m_pcFileSaveAction);
00461       pcMenu->addAction(m_pcFileSaveAsAction);
00462       m_pcFileSeparateRecentAction = pcMenu->addSeparator();
00463       for (int i = 0; i < MAX_RECENT_FILES; ++i) {
00464          pcMenu->addAction(m_pcFileOpenRecentAction[i]);
00465       }
00466       QToolBar* pcToolBar = addToolBar(tr("File"));
00467       pcToolBar->setObjectName("FileToolBar");
00468       pcToolBar->setIconSize(QSize(32,32));
00469       pcToolBar->addAction(m_pcFileNewAction);
00470       pcToolBar->addAction(m_pcFileOpenAction);
00471       pcToolBar->addAction(m_pcFileSaveAction);
00472       UpdateRecentFiles();
00473    }
00474 
00475    /****************************************/
00476    /****************************************/
00477 
00478    void CQTOpenGLLuaMainWindow::CreateEditActions() {
00479       QIcon cEditUndoIcon;
00480       cEditUndoIcon.addPixmap(QPixmap(m_pcMainWindow->GetIconDir() + "/undo.png"));
00481       m_pcEditUndoAction = new QAction(cEditUndoIcon, tr("&Undo"), this);
00482       m_pcEditUndoAction->setToolTip(tr("Undo last operation"));
00483       m_pcEditUndoAction->setStatusTip(tr("Undo last operation"));
00484       m_pcEditUndoAction->setShortcut(QKeySequence::Undo);
00485       connect(m_pcEditUndoAction, SIGNAL(triggered()),
00486               m_pcCodeEditor, SLOT(undo()));
00487       QIcon cEditRedoIcon;
00488       cEditRedoIcon.addPixmap(QPixmap(m_pcMainWindow->GetIconDir() + "/redo.png"));
00489       m_pcEditRedoAction = new QAction(cEditRedoIcon, tr("&Redo"), this);
00490       m_pcEditRedoAction->setToolTip(tr("Redo last operation"));
00491       m_pcEditRedoAction->setStatusTip(tr("Redo last operation"));
00492       m_pcEditRedoAction->setShortcut(QKeySequence::Redo);
00493       connect(m_pcEditRedoAction, SIGNAL(triggered()),
00494               m_pcCodeEditor, SLOT(redo()));
00495       QIcon cEditCopyIcon;
00496       cEditCopyIcon.addPixmap(QPixmap(m_pcMainWindow->GetIconDir() + "/copy.png"));
00497       m_pcEditCopyAction = new QAction(cEditCopyIcon, tr("&Copy"), this);
00498       m_pcEditCopyAction->setToolTip(tr("Copy selected text into clipboard"));
00499       m_pcEditCopyAction->setStatusTip(tr("Copy selected text into clipboard"));
00500       m_pcEditCopyAction->setShortcut(QKeySequence::Copy);
00501       connect(m_pcEditCopyAction, SIGNAL(triggered()),
00502               m_pcCodeEditor, SLOT(copy()));
00503       QIcon cEditCutIcon;
00504       cEditCutIcon.addPixmap(QPixmap(m_pcMainWindow->GetIconDir() + "/cut.png"));
00505       m_pcEditCutAction = new QAction(cEditCutIcon, tr("&Cut"), this);
00506       m_pcEditCutAction->setToolTip(tr("Move selected text into clipboard"));
00507       m_pcEditCutAction->setStatusTip(tr("Move selected text into clipboard"));
00508       m_pcEditCutAction->setShortcut(QKeySequence::Cut);
00509       connect(m_pcEditCutAction, SIGNAL(triggered()),
00510               m_pcCodeEditor, SLOT(cut()));
00511       QIcon cEditPasteIcon;
00512       cEditPasteIcon.addPixmap(QPixmap(m_pcMainWindow->GetIconDir() + "/paste.png"));
00513       m_pcEditPasteAction = new QAction(cEditPasteIcon, tr("&Paste"), this);
00514       m_pcEditPasteAction->setToolTip(tr("Paste text from clipboard"));
00515       m_pcEditPasteAction->setStatusTip(tr("Paste text from clipboard"));
00516       m_pcEditPasteAction->setShortcut(QKeySequence::Paste);
00517       connect(m_pcEditPasteAction, SIGNAL(triggered()),
00518               m_pcCodeEditor, SLOT(paste()));
00519       // QIcon cEditFindIcon;
00520       // cEditFindIcon.addPixmap(QPixmap(m_pcMainWindow->GetIconDir() + "/find.png"));
00521       // m_pcEditFindAction = new QAction(cEditFindIcon, tr("&Find/Replace"), this);
00522       // m_pcEditFindAction->setToolTip(tr("Find/replace text"));
00523       // m_pcEditFindAction->setStatusTip(tr("Find/replace text"));
00524       // m_pcEditFindAction->setShortcut(QKeySequence::Find);
00525       // connect(m_pcEditFindAction, SIGNAL(triggered()),
00526       //         this, SLOT(Find()));
00527       QMenu* pcMenu = menuBar()->addMenu(tr("&Edit"));
00528       pcMenu->addAction(m_pcEditUndoAction);
00529       pcMenu->addAction(m_pcEditRedoAction);
00530       pcMenu->addSeparator();
00531       pcMenu->addAction(m_pcEditCopyAction);
00532       pcMenu->addAction(m_pcEditCutAction);
00533       pcMenu->addAction(m_pcEditPasteAction);
00534       // pcMenu->addSeparator();
00535       // pcMenu->addAction(m_pcEditFindAction);
00536       QToolBar* pcToolBar = addToolBar(tr("Edit"));
00537       pcToolBar->setObjectName("EditToolBar");
00538       pcToolBar->setIconSize(QSize(32,32));
00539       pcToolBar->addAction(m_pcEditUndoAction);
00540       pcToolBar->addAction(m_pcEditRedoAction);
00541       pcToolBar->addSeparator();
00542       pcToolBar->addAction(m_pcEditCopyAction);
00543       pcToolBar->addAction(m_pcEditCutAction);
00544       pcToolBar->addAction(m_pcEditPasteAction);
00545       // pcToolBar->addAction(m_pcEditFindAction);
00546    }
00547 
00548    /****************************************/
00549    /****************************************/
00550 
00551    void CQTOpenGLLuaMainWindow::CreateCodeActions() {
00552       QIcon cCodeExecuteIcon;
00553       cCodeExecuteIcon.addPixmap(QPixmap(m_pcMainWindow->GetIconDir() + "/execute.png"));
00554       m_pcCodeExecuteAction = new QAction(cCodeExecuteIcon, tr("&Execute"), this);
00555       m_pcCodeExecuteAction->setToolTip(tr("Execute code"));
00556       m_pcCodeExecuteAction->setStatusTip(tr("Execute code"));
00557       m_pcCodeExecuteAction->setShortcut(tr("Ctrl+E"));
00558       connect(m_pcCodeExecuteAction, SIGNAL(triggered()),
00559               this, SLOT(Execute()));
00560       QMenu* pcMenu = menuBar()->addMenu(tr("&Code"));
00561       pcMenu->addAction(m_pcCodeExecuteAction);
00562       QToolBar* pcToolBar = addToolBar(tr("Code"));
00563       pcToolBar->setObjectName("CodeToolBar");
00564       pcToolBar->setIconSize(QSize(32,32));
00565       pcToolBar->addAction(m_pcCodeExecuteAction);
00566    }
00567 
00568    /****************************************/
00569    /****************************************/
00570 
00571    void CQTOpenGLLuaMainWindow::OpenFile(const QString& str_path) {
00572       QFile cFile(str_path);
00573       if(! cFile.open(QFile::ReadOnly | QFile::Text)) {
00574          QMessageBox::warning(this, tr("ARGoS v" ARGOS_VERSION "-" ARGOS_RELEASE " - Lua Editor"),
00575                               tr("Cannot read file %1:\n%2.")
00576                               .arg(str_path)
00577                               .arg(cFile.errorString()));
00578          return;
00579       }
00580       QApplication::setOverrideCursor(Qt::WaitCursor);
00581       m_pcCodeEditor->setPlainText(cFile.readAll());
00582       QApplication::restoreOverrideCursor();
00583       SetCurrentFile(str_path);
00584       statusBar()->showMessage(tr("File loaded"), 2000);
00585    }
00586 
00587    /****************************************/
00588    /****************************************/
00589 
00590    bool CQTOpenGLLuaMainWindow::SaveFile(const QString& str_path) {
00591       QFile cFile(str_path);
00592       if(! cFile.open(QFile::WriteOnly | QFile::Text)) {
00593          QMessageBox::warning(this, tr("ARGoS v" ARGOS_VERSION "-" ARGOS_RELEASE " - Lua Editor"),
00594                               tr("Cannot write file %1:\n%2.")
00595                               .arg(str_path)
00596                               .arg(cFile.errorString()));
00597          return false;
00598       }
00599       QTextStream cOut(&cFile);
00600       QApplication::setOverrideCursor(Qt::WaitCursor);
00601       cOut << m_pcCodeEditor->toPlainText();
00602       QApplication::restoreOverrideCursor();
00603       SetCurrentFile(str_path);
00604       statusBar()->showMessage(tr("File saved"), 2000);
00605       return true;
00606    }
00607 
00608    /****************************************/
00609    /****************************************/
00610 
00611    void CQTOpenGLLuaMainWindow::SetCurrentFile(const QString& str_path) {
00612       m_strFileName = str_path;
00613       QString strShownName;
00614       if(m_strFileName.isEmpty()) {
00615          strShownName = "untitled";
00616       }
00617       else {
00618          strShownName = StrippedFileName(m_strFileName);
00619       }
00620       setWindowTitle(tr("%1[*] - ARGoS v" ARGOS_VERSION "-" ARGOS_RELEASE " - Lua Editor").arg(strShownName));
00621       if(!m_strFileName.isEmpty()) {
00622          m_pcCodeEditor->document()->setModified(false);
00623          setWindowModified(false);
00624          QSettings cSettings;
00625          cSettings.beginGroup("LuaEditor");
00626          QStringList listFiles = cSettings.value("recent_files").toStringList();
00627          listFiles.removeAll(m_strFileName);
00628          listFiles.prepend(m_strFileName);
00629          while(listFiles.size() > MAX_RECENT_FILES) {
00630             listFiles.removeLast();
00631          }
00632          cSettings.setValue("recent_files", listFiles);
00633          cSettings.endGroup();
00634          UpdateRecentFiles();
00635       }
00636       else {
00637          m_pcCodeEditor->document()->setModified(true);
00638          setWindowModified(true);
00639       }
00640    }
00641 
00642    /****************************************/
00643    /****************************************/
00644 
00645    void CQTOpenGLLuaMainWindow::CheckLuaStatus(int n_step) {
00646       int nRow = 0;
00647       m_pcLuaMessageTable->clearContents();
00648       m_pcLuaMessageTable->setRowCount(m_vecControllers.size());
00649       for(size_t i = 0; i < m_vecControllers.size(); ++i) {
00650          if(! m_vecControllers[i]->IsOK()) {
00651             SetMessage(nRow,
00652                        QString::fromStdString(m_vecControllers[i]->GetId()),
00653                        QString::fromStdString(m_vecControllers[i]->GetErrorMessage()));
00654             ++nRow;
00655          }
00656       }
00657       m_pcLuaMessageTable->setRowCount(nRow);
00658       if(nRow > 0) {
00659          m_pcMainWindow->SuspendExperiment();
00660       }
00661       else {
00662          m_pcLuaMsgDock->hide();
00663       }
00664    }
00665 
00666    /****************************************/
00667    /****************************************/
00668 
00669    void CQTOpenGLLuaMainWindow::HandleMsgTableSelection() {
00670       QList<QTableWidgetItem*> listSel = m_pcLuaMessageTable->selectedItems();
00671       if(! listSel.empty()) {
00672          int nLine = listSel[1]->data(Qt::DisplayRole).toInt();
00673          QTextCursor cCursor = m_pcCodeEditor->textCursor();
00674          int nCurLine = cCursor.blockNumber();
00675          if(nCurLine < nLine) {
00676             cCursor.movePosition(QTextCursor::NextBlock,
00677                                  QTextCursor::MoveAnchor,
00678                                  nLine - nCurLine - 1);
00679          }
00680          else if(nCurLine > nLine) {
00681             cCursor.movePosition(QTextCursor::PreviousBlock,
00682                                  QTextCursor::MoveAnchor,
00683                                  nCurLine - nLine + 1);
00684          }
00685          cCursor.movePosition(QTextCursor::StartOfBlock);
00686          m_pcCodeEditor->setTextCursor(cCursor);
00687          m_pcCodeEditor->setFocus();
00688       }
00689    }
00690 
00691    /****************************************/
00692    /****************************************/
00693 
00694    void CQTOpenGLLuaMainWindow::HandleEntitySelection(size_t un_index) {
00695       CComposableEntity* pcSelectedEntity = dynamic_cast<CComposableEntity*>(CSimulator::GetInstance().GetSpace().GetRootEntityVector()[un_index]);
00696       if(pcSelectedEntity != NULL) {
00697          bool bFound = false;
00698          m_unSelectedRobot = 0;
00699          while(!bFound && m_unSelectedRobot < m_vecRobots.size()) {
00700             if(m_vecRobots[m_unSelectedRobot] == pcSelectedEntity) {
00701                bFound = true;
00702             }
00703             else {
00704                ++m_unSelectedRobot;
00705             }
00706          }
00707          if(bFound &&
00708             m_vecControllers[m_unSelectedRobot]->GetLuaState() != NULL) {
00709             CQTOpenGLLuaStateTreeVariableModel* pcVarModel =
00710                new CQTOpenGLLuaStateTreeVariableModel(m_vecControllers[m_unSelectedRobot]->GetLuaState(),
00711                                                       false,
00712                                                       m_pcLuaVariableTree);
00713             pcVarModel->Refresh();
00714             connect(&(m_pcMainWindow->GetOpenGLWidget()), SIGNAL(StepDone(int)),
00715                     pcVarModel, SLOT(Refresh(int)));
00716             connect(m_pcMainWindow, SIGNAL(ExperimentReset()),
00717                     pcVarModel, SLOT(Refresh()));
00718             connect(pcVarModel, SIGNAL(modelReset()),
00719                     this, SLOT(VariableTreeChanged()),
00720                     Qt::QueuedConnection);
00721             m_pcLuaVariableTree->setModel(pcVarModel);
00722             m_pcLuaVariableTree->setRootIndex(pcVarModel->index(0, 0));
00723             m_pcLuaVariableTree->expandAll();
00724             m_pcLuaVariableDock->show();
00725             CQTOpenGLLuaStateTreeFunctionModel* pcFunModel =
00726                new CQTOpenGLLuaStateTreeFunctionModel(m_vecControllers[m_unSelectedRobot]->GetLuaState(),
00727                                                       true,
00728                                                       m_pcLuaFunctionTree);
00729             pcFunModel->Refresh();
00730             connect(&(m_pcMainWindow->GetOpenGLWidget()), SIGNAL(StepDone(int)),
00731                     pcFunModel, SLOT(Refresh(int)));
00732             connect(m_pcMainWindow, SIGNAL(ExperimentReset()),
00733                     pcFunModel, SLOT(Refresh()));
00734             connect(pcFunModel, SIGNAL(modelReset()),
00735                     this, SLOT(FunctionTreeChanged()),
00736                     Qt::QueuedConnection);
00737             m_pcLuaFunctionTree->setModel(pcFunModel);
00738             m_pcLuaFunctionTree->setRootIndex(pcFunModel->index(0, 0));
00739             m_pcLuaFunctionTree->expandAll();
00740             m_pcLuaFunctionDock->show();
00741          }
00742       }
00743    }
00744 
00745    /****************************************/
00746    /****************************************/
00747 
00748    void CQTOpenGLLuaMainWindow::HandleEntityDeselection(size_t) {
00749       disconnect(&(m_pcMainWindow->GetOpenGLWidget()), SIGNAL(StepDone(int)),
00750                  m_pcLuaVariableTree->model(), SLOT(Refresh(int)));
00751       disconnect(m_pcMainWindow, SIGNAL(ExperimentReset()),
00752                  m_pcLuaVariableTree->model(), SLOT(Refresh()));
00753       disconnect(m_pcLuaVariableTree->model(), SIGNAL(modelReset()),
00754                  this, SLOT(VariableTreeChanged()));
00755       m_pcLuaVariableDock->hide();
00756       delete m_pcLuaVariableTree->model();
00757       m_pcLuaVariableTree->setModel(NULL);
00758       disconnect(&(m_pcMainWindow->GetOpenGLWidget()), SIGNAL(StepDone(int)),
00759                  m_pcLuaFunctionTree->model(), SLOT(Refresh(int)));
00760       disconnect(m_pcMainWindow, SIGNAL(ExperimentReset()),
00761                  m_pcLuaFunctionTree->model(), SLOT(Refresh()));
00762       disconnect(m_pcLuaFunctionTree->model(), SIGNAL(modelReset()),
00763                  this, SLOT(FunctionTreeChanged()));
00764       m_pcLuaFunctionDock->hide();
00765       delete m_pcLuaFunctionTree->model();
00766       m_pcLuaFunctionTree->setModel(NULL);
00767    }
00768 
00769    /****************************************/
00770    /****************************************/
00771 
00772    void CQTOpenGLLuaMainWindow::VariableTreeChanged() {
00773       m_pcLuaVariableTree->setRootIndex(m_pcLuaVariableTree->model()->index(0, 0));
00774       m_pcLuaVariableTree->expandAll();
00775    }
00776 
00777    /****************************************/
00778    /****************************************/
00779 
00780    void CQTOpenGLLuaMainWindow::FunctionTreeChanged() {
00781       m_pcLuaFunctionTree->setRootIndex(m_pcLuaFunctionTree->model()->index(0, 0));
00782       m_pcLuaFunctionTree->expandAll();
00783    }
00784 
00785    /****************************************/
00786    /****************************************/
00787 
00788    void CQTOpenGLLuaMainWindow::UpdateRecentFiles() {
00789       QSettings cSettings;
00790       cSettings.beginGroup("LuaEditor");
00791       QStringList listFiles = cSettings.value("recent_files").toStringList();
00792       int nRecentFiles = qMin(listFiles.size(), (int)MAX_RECENT_FILES);
00793       for(int i = 0; i < nRecentFiles; ++i) {
00794          m_pcFileOpenRecentAction[i]->setText(tr("&%1 %2").arg(i+1).arg(StrippedFileName(listFiles[i])));
00795          m_pcFileOpenRecentAction[i]->setData(listFiles[i]);
00796          m_pcFileOpenRecentAction[i]->setVisible(true);
00797       }
00798       for(int i = nRecentFiles; i < MAX_RECENT_FILES; ++i) {
00799          m_pcFileOpenRecentAction[i]->setVisible(false);
00800       }
00801       m_pcFileSeparateRecentAction->setVisible(nRecentFiles > 0);
00802       cSettings.endGroup();
00803    }
00804 
00805    /****************************************/
00806    /****************************************/
00807 
00808    void CQTOpenGLLuaMainWindow::SetMessage(int n_row,
00809                                            const QString& str_robot_id,
00810                                            const QString& str_message) {
00811       QStringList listFields = str_message.split(":",
00812                                                  QString::KeepEmptyParts,
00813                                                  Qt::CaseInsensitive);
00814       m_pcLuaMessageTable->setItem(
00815          n_row, 0,
00816          new QTableWidgetItem(str_robot_id));
00817       if(listFields.size() == 3) {
00818          m_pcLuaMessageTable->setItem(
00819             n_row, 1,
00820             new QTableWidgetItem(listFields[1]));
00821          m_pcLuaMessageTable->setItem(
00822             n_row, 2,
00823             new QTableWidgetItem(listFields[2]));
00824       }
00825       else if(listFields.size() == 4) {
00826          m_pcLuaMessageTable->setItem(
00827             n_row, 1,
00828             new QTableWidgetItem(listFields[2]));
00829          m_pcLuaMessageTable->setItem(
00830             n_row, 2,
00831             new QTableWidgetItem(listFields[3]));
00832       }
00833       else {
00834          m_pcLuaMessageTable->setItem(
00835             n_row, 2,
00836             new QTableWidgetItem(str_message));
00837       }
00838       m_pcLuaMsgDock->show();
00839    }
00840 
00841    /****************************************/
00842    /****************************************/
00843 
00844    QString CQTOpenGLLuaMainWindow::StrippedFileName(const QString& str_path) {
00845       return QFileInfo(str_path).fileName();
00846    }
00847 
00848    /****************************************/
00849    /****************************************/
00850 
00851    void CQTOpenGLLuaMainWindow::closeEvent(QCloseEvent* pc_event) {
00852       pc_event->ignore();
00853    }
00854 
00855    /****************************************/
00856    /****************************************/
00857 
00858 }
```

---

Generated on 10 Jul 2018 for ARGoS by 
 1.6.1
